# Unraveling the Intrinsic Mechanisms Controlling the Variations in Density, Sensitivity, and Thermal Decomposition of Typical Nitroguanidine Derivatives

**DOI:** 10.3390/molecules30214204

**Published:** 2025-10-28

**Authors:** Pengshan Geng, Songsong Guo, Xiaohong Wang, Chao Xing, Chenxi Qu, Jieyu Luan, Kewei Ding

**Affiliations:** 1 Xi’an Modern Chemistry Research Institute, Xi’an 710065, China; gengps2002@163.com (P.G.); 13980021896@163.com (S.G.); wxh_813@163.com (X.W.); 13965767618@163.com (C.X.); qcx204@163.com (C.Q.); luanjieyu@126.com (J.L.); 2 State Key Laboratory of Fluorine & Nitrogen Chemical, Xi’an 710065, China

**Keywords:** energetic materials, nitroguanidine derivatives, structure–property relationship, density functional theory

## Abstract

Nitroguanidine-type energetic materials have broad application prospects in the propellant field, and their derivative structures are numerous, with intricate changes in macro-level properties. However, due to the unclear inherent evolution mechanisms of these macro-level properties, the structural optimization of compounds and the iteration of application systems face difficulties. This work systematically investigates the variations in density, thermal decomposition, and sensitivity among nitroguanidine (NQ), 1-amino-2-nitroguanidine (ANQ), and 1-amino-2-nitroguanidinium nitrate (ANGN). Hirshfeld surface and bond dissociation energy analyses reveal that strengthened electrostatic and inductive interactions enhance the hydrogen bonding network in ANGN, leading to its higher density compared to NQ. In contrast, weakened electrostatic interactions in ANQ result in a less robust hydrogen bonding network and a correspondingly lower density. The sensitivity trend is consistently explained from both molecular and crystalline perspectives: an increasingly inhomogeneous electrostatic potential distribution, coupled with a higher frequency of O···O contacts, provides a coherent explanation for the experimental observations. Furthermore, the electron-withdrawing -NH_3_^+^ group in ANGN weakens the N–NO_2_ bond, reducing its bond dissociation energy and leading to the most intense NO_2_ mass spectral signal during thermal decomposition. ANQ exhibits the opposite behavior. A linear correlation (R^2^ = 0.92) is observed between the N–NO_2_ BDE and NO_2_ mass spectral intensity across NQ, ANQ, and ANGN. This study provides unique insights into the intrinsic mechanisms governing variations in the properties of nitroguanidine derivatives.

## 1. Introduction

Energetic materials (EMs) epitomize humanity’s quest to harness chemical energy at atomic scales [1,2,3,4]. Generally, the desired high performance can be achieved by using compounds with zero oxygen balance, which can maximize the release of energy without any waste, but these high-energy-density materials (HEDMs) also tend to be sensitive to to external stimuli [5,6,7,8]. The contrary behavior of the required parameters of HEDMs leads to the conclusion that not only the molecular design but also the crystallographic design should be considered to find a balance between performance and safety for new energetic materials.

**Figure 1:**
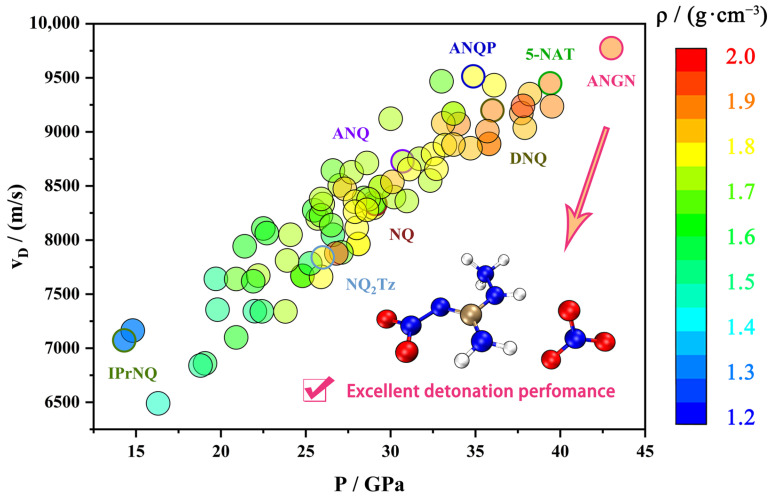
A comparison of density and detonation performance between nitroguanidine and its derivatives.

Nitroguanidine (NQ) is a high explosive known for its extremely low sensitivity to mechanical stimuli and shock waves [9,10,11]. The presence of the strongly electron-accepting nitroimine moiety makes it easily functionalized by exchanging the amine groups or substituting the hydrogen atoms of these groups. Consequently, nitroguanidine is recognized as a good building block. In addition, these groups that are both good donors and acceptors of the electron pair enable the creation of extensive networks of hydrogen bonds, which contributes to lowering the sensitivity to all types of initiating stimuli. NQ maintains insensitivity while also having high detonation performance and is therefore used as a component of gunpowder, rocket propellants, and high explosives. Figure 1 shows the density, detonation velocity, and pressure of NQ and its 85 derivatives [11,12]. There are many great derivatives, such as 1-amino-2-nitroguanidine (ANQ), with surprisingly abundant reactivity; 1-amino-2-nitroguanidium pentazolate (ANQP) [13], with almost the highest density within the non-metallic pentazolate salts; and 1-amino-2-nitroguanidinium nitrate (ANGN), with almost the highest detonation velocity and pressure (V_D_ = 9775 m·s^−1^, P = 43.0 GPa) among the derivatives of NQ, making it promising for application in gun propellants [14].

As shown above, energetic salts are regarded as a possible approach to the search for HEDMs, because they often possess advantages over non-ionic analogs, such as higher density, lower vapor pressure, and better modularity [15,16,17]. The development of advanced energetic materials is driving a paradigm shift from traditional organic synthesis toward modular, multi-ion assembly strategies. Although ANGN was discovered in 2012 and exhibited outstanding detonation performance [18], previous work has ignored the changes in the properties of ANGN during the synthesis process. Therefore, the study of its structure–property relationships, especially the reasons for the structure and property changes in ANGN and its synthetic precursors, is important and necessary.

In this work, we synthesized NQ, ANQ, and ANGN; discussed their differences in structure, thermal decomposition, and sensitivity; and explained the changes in density, sensitivity, and thermal decomposition by combining theoretical methods with structural investigations to analyze their intrinsic mechanisms.

## 2. Results and Discussion

### 2.1. Synthesis

**Scheme 1:**
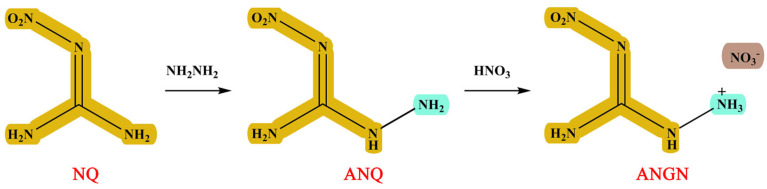
Synthesis route of ANQ and ANGN.

1-Amino-2-nitroguanidine (ANQ) and 1-amino-2-nitroguanidinium nitrate (ANGN) were synthesized using Scheme 1. They have been documented in the literature and were fully characterized by infrared and NMR spectra [18].

### 2.2. Differences in Molecular and Crystal Structure

From the perspective of molecular structure, ANGN should have a higher density than ANQ, because the combination of H^+^ and the NH_2_ group decreases the Pauli repulsion from the lone-pair electron of the NH_2_ group, and the introduction of nitrate anions improves the efficiency of crystal packing.

**Figure 2:**
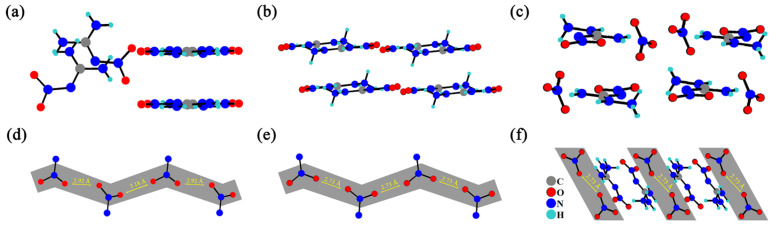
The crystal packing structures of NQ (**a**), ANQ (**b**), and ANGN (**c**). The distance between the two nearest oxygen atoms in NQ (**d**), ANQ (**e**), and ANGN (**f**).

**Figure 3:**
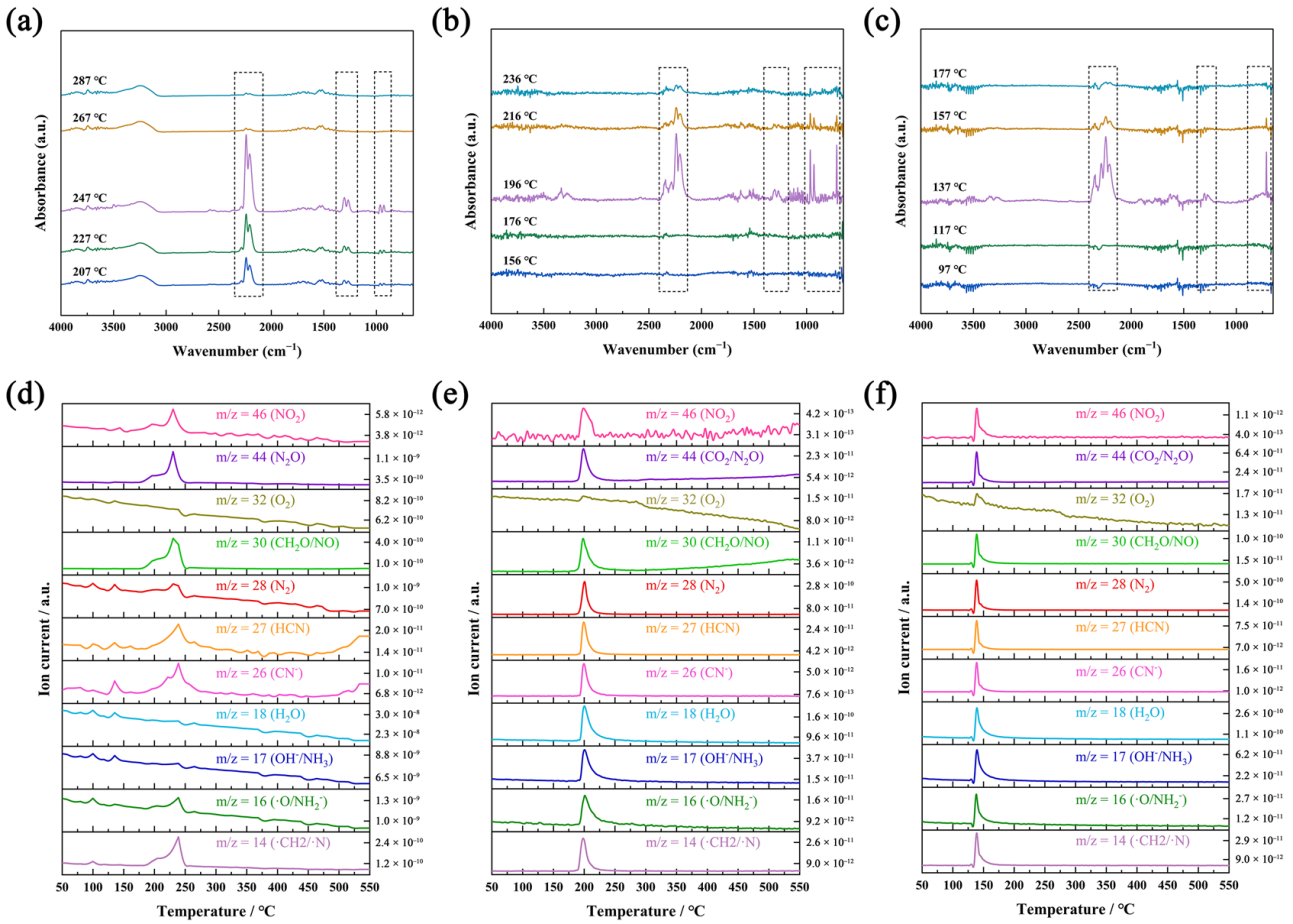
Infrared spectra at different heating temperatures of gas-phase products from the decomposition of NQ (**a**), ANQ (**b**), and ANGN (**c**). Ion current intensity of NQ (**d**), ANQ (**e**), and ANGN’s (**f**) gas-phase product with temperature changes.

From the perspective of crystal structure, their crystal packing structures are shown in Figure 2a–c, where these close contacts of nitrate anions are clearly observed. Figure 3d–f show that the distances between the two nearest oxygen atoms in NQ, ANQ, and ANGN are 2.92, 2.73, and 2.73 Å, respectively. The close distance between oxygen atoms indicates that ANQ and ANGN would be more sensitive than NQ, as the swing of a single nitro group or nitrate anion toward mechanical stimulus will probably induce a whole-chain response and result in lattice deformation. Under the same conditions, ANQ with a layered structure will be more insensitive than ANGN.

### 2.3. Differences in Thermal Decomposition Behavior

TG-DSC-IR-MS tests were used to investigate the thermal behavior of NQ, ANQ, and ANGN. Their TG curves all exhibit a sharp decline at 247, 196, and 148 °C, respectively, due to remarkably rapid decomposition, generating gas and resulting in a sudden weight loss (Figure S8). They are highly consistent with the exothermic peaks appearing at the same position in the DSC curves. In the infrared spectra (Figure 3a–c), some obvious characteristic peaks were detected near their peak temperature, indicating the formation of gas-phase products that should be NO_2_ (1593~1635 cm^−1^), N_2_O (2201~2239 cm^−1^), CO_2_ (667 and 2360 cm^−1^), H_2_O (3500~4000 cm^−1^), and NH_3_ (930 and 960 cm^−1^). The infrared characteristic peaks of all gas products gradually disappear with increasing temperature after the peak temperature. In addition, Figure 3d–f illustrate the variation curves of ion current intensity with the temperature of gas-phase products from the thermal decomposition of NQ, ANQ, and ANGN. The primarily detected ion fragments are *m*/*z* = 46 (NO_2_), *m*/*z* = 44 (CO_2_ and N_2_O), *m*/*z* = 32 (O_2_), *m*/*z* = 30 (CH_2_O and NO), *m*/*z* = 28 (N_2_), *m*/*z* = 27 (HCN), *m*/*z* = 26 (CN^−^), *m*/*z* = 18 (H_2_O), *m*/*z* = 17 (OH^−^ and NH_3_), *m*/*z* = 16 (O and NH_2_^−^), and *m*/*z* = 14 (CH_2_ and·N). The three most intense fragments are N_2_O, CH_2_O/NO, and CH_2_/N for NQ; N_2_, H_2_O, and OH^−^/NH_3_ for ANQ; and N_2_, H_2_O, and CH_2_O/NO for ANGN. Combined with their infrared spectrum at peak temperature, the top four intense gas-phase products of NQ are N_2_O and NH_3_, those of ANQ are N_2_, N_2_O, CO_2_, NH_3_, and H_2_O, and those of ANGN are N_2_, N_2_O, CO_2_, H_2_O, and NH_3_. The difference in gas decomposition products suggests the existence of distinct decomposition mechanisms.

### 2.4. Differences in Physicochemical Properties

Based on the density and heat of formation literature, the detonation properties of NQ, ANQ, ANGN, and reference energetic materials were calculated by the EXPLO5 (v6.04) program. As shown in Table 1, the calculated detonation velocity and pressure of ANGN are 9438 m·s^−1^ and 40.2 GPa, significantly outperforming RDX (8801 m·s^−1^, 33.6 GPa) and HMX (9193 m·s^−1^, 37.8 GPa). Meanwhile, ANGN still has a high density (1.86 g·cm^−3^ at 298 K) and low sensitivities (10 J for IS, 120 N for FS). In addition, we noticed that the thermal decomposition from NQ to ANGN had declined, while the sensitivity had increased. To find the reason for this change, the influences of the introduced groups were assessed through various theoretical analysis methods using density functional theory of molecular and crystal structure.

### 2.5. The Influence of the Introduced Group on Molecular Properties

**Figure 4:**
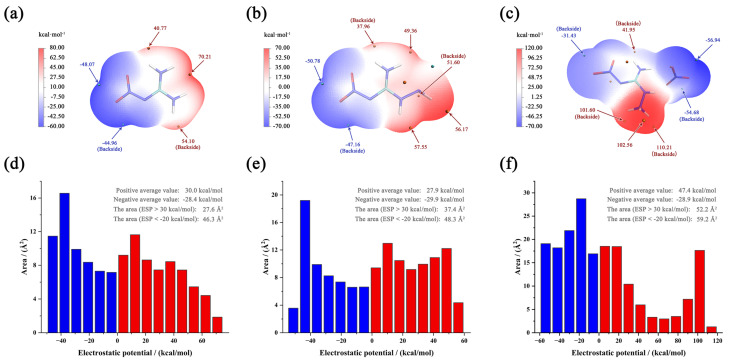
ESP-mapped molecular vdW surfaces of NQ (**a**), ANQ (**b**), and ANGN (**c**). The area distribution maps of ESP for NQ (**d**), ANQ (**e**), and ANGN (**f**).

Electrostatic potential (ESP) is often used to understand changes in sensitivities and visualize the bond strength variation [22]. Specifically, molecules that exhibit extensive areas with larger and stronger positive electrostatic potentials tend to exhibit increased impact sensitivities. This relationship can be attributed to the fact that regions of high positive potential can attract negatively charged entities or impactors, facilitating the initiation of explosive reactions [23]. The visualization of ESP for three compounds is shown in Figure 4a–c. For NQ and ANQ, the positive range and values are approximately equal to the negative range and values. Their differences between maximum and minimum are 118.28 and 108.33 kJ·mol^−1^, respectively. The values are very close and cannot support an accurate judgment. For ANGN, ESP reveals critical charge polarization: (1) maximum positive potential (110.21 kJ·mol^−1^) localized at the NH_3_ group; (2) minimum negative potential (−56.94 kJ·mol^−1^) distributed over NO_3_^−^ anions.

Besides the ESP surface, an area distribution map of ESP is also an effective analysis method. The maps of the three compounds are shown in Figure 4d–f. Due to the polarization distribution and the high average value of the positive potential, ANGN remains the most sensitive. For NQ and ANQ, their positive and negative average values are almost equal, but ANQ has a larger area with positive (>30 kJ·mol^−1^) and negative (<−20 kJ·mol^−1^) potential, with values of 37.4 and 48.3 Å^2^, respectively, compared to 27.6 and 46.3 Å^2^ for NQ. This is attributed to the electron-donating effect of the introduced -NH_2_ group. Considering that excessive regions of high electrostatic potential can lead to potential intermolecular repulsion, NQ should be the most insensitive. Therefore, the order of sensitivity should be ANGN > ANQ > NQ, and the same trend is present in experimental observations.

**Figure 5:**
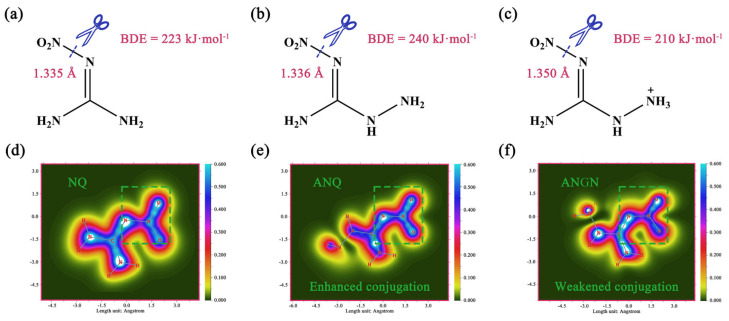
Calculated BDE values of the weakest bond in the molecules NQ (**a**), ANQ (**b**), and ANGN (**c**). The electron delocalization paths (1.0 Bohr above the XY plane) of NQ (**d**), ANQ (**e**), and ANGN (**f**).

Besides the calculation of ESP, bond dissociation enthalpy (BDE) is considered the most important factor in pyrogenic decomposition for the possible trigger bond that will break first and can be used to assess the stability of a material [24]. In this study, the BDEs were calculated from their crystal structure data, and the values are depicted in Figure 5. The values of the BDEs from NQ to ANGN are 223, 240, and 210 kJ·mol^−1^, which indicates that the N–NO_2_ bond of ANQ is the most stable and that of ANGN is the most unstable. In the above MS analysis, the contents of NO_2_ in NQ, ANQ, and ANGN were determined to be 2.378 × 10^−7^, 3.558 ×10^−8^, and 2.847 × 10^−7^ mol^−1^ at the peak temperature of decomposition, and the linear fitting coefficient R^2^ between it and BDE is 0.92. The bond dissociation energy accounts well for the differences in NO_2_ yield among NQ, ANQ, and ANGN. Since NO_2_ is produced from N–NO_2_ bond cleavage, a weaker bond results in a higher yield. Also, this result gives the specific presentation of BDE during the decomposition process.

This change in BDE cannot be explained accurately by the bond length, and therefore localized orbital locator-π (LOL-π) was employed to evaluate the conjugation degree of the three molecules. The electron-detonating group (-NH_2_) enhances the conjugation of the nitramino group, but the electron-withdrawing group (-NH_3_^+^) weakens it. Obviously, the introduced group changes the electron structure of the molecule and influences the conjugation. This result indicates that the order of thermal stability is ANQ > NQ > ANGN. However, the order of decomposition temperature is, in fact, NQ > ANQ > ANGN. In the HOMO-LUMO analysis (Figure S10), NQ has the largest energy gap of 8.18 eV, with ANGN possessing the smallest at 5.77 eV, which can provide a reasonable explanation for the variation in the thermal decomposition temperatures among the three compounds. Undoubtedly, there should be some lower reaction barriers present for NQ, ANQ, and ANGN, and they can be decomposed through other pathways.

### 2.6. The Influence of the Introduced Group on the Crystal Structure

**Figure 6:**
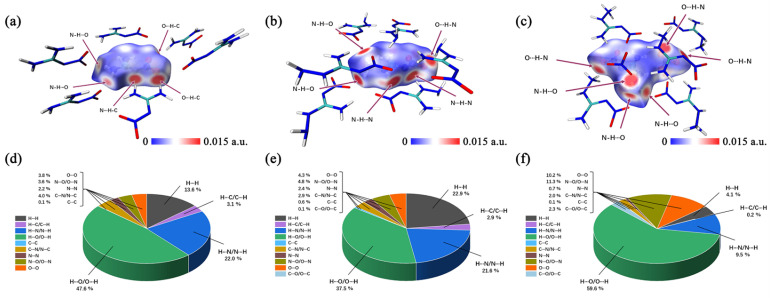
Hirshfeld surface analysis of NQ (**a**), ANQ (**b**), and ANGN (**c**). Single-atom contact percentage of NQ (**d**), ANQ (**e**), and ANGN (**f**).

The non-conventional O···O interaction is a very important close-contact interaction. In most cases, a high frequency of O···O contacts indicates a high sensitivity, because more nitro groups are exposed on the molecular surface and that increases the risk of explosion due to the exceeding repulsion via an interlayer sliding [23]. Thus, Figure 6d–f clearly show that ANGN is the most sensitive compound. With 10.2% of O···O contacts, ANGN has the most of those contacts, which are contributed by these introduced NO_3_^−^ anions, compared to ANQ with 4.3% and NQ with 3.8%. The frequency of O···O contacts is perfectly consistent with the order of experimental sensitivity.

Except for O···O contacts, contact percentage maps identify hydrogen bonding interactions (N···H/H···N/O···H/H···O/C···H/H···C), collectively constituting 72.7%, 62.0%, and 69.3% of total intermolecular contacts (Figure 6d–f) in NQ, ANQ, and ANGN, respectively. Meanwhile, Figure 6a–c and the IGMH isosurfaces (Figure S12a–c) provide the distinct surface contact patterns, and the 2D fingerprint plots (Figure S11) also exhibit characteristic wing-shaped projections of hydrogen bonding interactions. As shown in Figure S12d–f, both ANQ and ANGN exhibit a distinct peak in the range of 0.01 to 0.02, which is absent in NQ. The presence of this peak indicates repulsive interactions between the central molecule and its surroundings, correlating with an excessive area of high electrostatic potential on the molecular surface. Furthermore, the magnitude of these repulsive peaks (NQ < ANQ < ANGN) suggests that ANGN has the highest sensitivity, while NQ has the lowest. This result is consistent with and reinforces the conclusions from the preceding electrostatic potential and O···O interaction analyses.

Although the Hirshfeld surface shows the site and percentage of hydrogen bonds, its effect on properties is unclear. To obtain the accurate energies of their hydrogen bonds, EDA (energy decomposition analysis) was employed, and the results are depicted in Table S1. EDA is an important class of method for exploring the nature of interaction between fragments in a chemical system. It can decompose the interaction energy into different physical components to understand the factors that play key roles in the interaction. This work uses an EDA strategy based on dispersion-corrected density functional theory, called sobEDA. The sobEDAw method used in this work was applied for the study of weakly interacting systems [25]. The total hydrogen bond energies for NQ, ANQ, and ANGN are −79.45, −58.93, and −227.96 kJ·mol^−1^, indicating that the 3D hydrogen bonding network of ANGN is the hardest and that of ANQ is the softest. Therefore, the enhancing effect of the hydrogen bonding network on density should be strongest for ANGN, and weakest for ANQ, which effectively explains the order of their crystal density (ANGN > NQ > ANQ).

**Figure 7:**
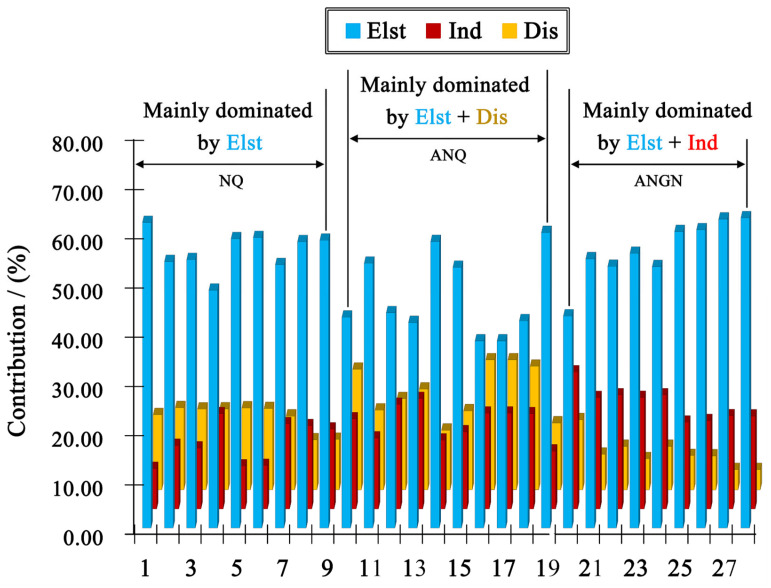
Contribution percentage plot of the sobEDAw-derived constructive electrostatic (Elst), induction (Ind), and dispersion (Dis) interactions within the hydrogen bond formation for NQ, ANQ, and ANGN.

From Figure 7 and Table S1, one can find that the electrostatic contribution always plays a major role, while the stabilization of the dimers is also effectively assisted by induction or dispersion interactions. For ANQ, the system remains neutral, with electrostatic decreases and exchange–repulsion increases, making hydrogen bonds exhibit low bond energy (<10 kcal·mol^−1^). For ANGN, the system changes from neutral to charged, and electrostatic and induction increase sharply, making hydrogen bonds exhibit high bond energy (>20 kcal·mol^−1^). Consequently, in ANGN, the charged nature strengthens the hydrogen bonding network through increased electrostatic and inductive interactions, thereby leading to a higher density. In contrast, for ANQ, the decreased electrostatic interactions, which may be associated with a reduced maximum electrostatic potential at the amino group, result in a weakened hydrogen bonding network, achieving a lower density. Furthermore, the 3D hydrogen bonding network can make a compound more sensitive, since an interlayer slide strongly alters these stabilizing interactions. However, the replacement of hard O···O interactions with softer N···H or O···H interactions often leads to a better absorption of mechanical stimuli in a material [23]. In addition, studies have shown that when a molecule acts as a hydrogen bond acceptor, it leads to an increase in positive electrostatic potential, thereby enhancing detonation sensitivity [26]. In this case, the proportion of interactions where the molecule acts as a hydrogen bond acceptor is as high as 66.7% for ANGN, compared to 60% for ANQ and 55.6% for NQ. Thus, from two perspectives on the impact of hydrogen bonding on sensitivity, the expected sensitivity order is ANGN > ANQ > NQ, consistent with the results obtained from both the ESP and O···O contact analyses.

## 3. Materials and Methods

### 3.1. Caution

Although unexpected explosions and hazards were not encountered during this study, small-scale and best safety practices (explosion-proof baffle, face shield, and leather gloves) are strongly encouraged.

### 3.2. General Procedures

The ^1^H and ^13^C NMR spectra were obtained on a Bruker-AV 500 (Billerica, MA, USA, 500, 126, and 36 MHz, respectively) using DMSO as the internal standard (^1^H NMR: 2.50 ppm; ^13^C NMR: 39.52 ppm). Infrared spectroscopy was performed using an infrared spectrometer (Thermo Scientific (Waltham, MA, USA), ATR50). High-resolution mass spectrometry was carried out using a Netzsch (Selb, Germany), QMS403 mass spectrometer using electrospray ionization. DSC-TG-MS-IR analysis was performed on a simultaneous thermal analyzer (NETZSCH (Selb, Germany) STA449F3 STA). 

### 3.3. Synthesis of Derivatives

#### 3.3.1. 2-Nitroguanidine (NQ)

A 100 mL flask was charged with concentrated H_2_SO_4_ (20 mL) and cooled in an ice bath. Guanidine nitrate (12.2 g) was added portion-wise. After completion, the mixture was heated at 50 °C (oil bath) with stirring for 1 h, cooled to room temperature, and quenched into ice water (80 mL). The copious white precipitate was collected via suction filtration, washed with H_2_O, and air-dried to afford nitroguanidine (8.2 g).

#### 3.3.2. 1-Amino-2-nitroguanidine (ANQ)

In a 100 mL reaction flask, nitroguanidine (5.2 g) and water (60 mL) were charged. The mixture was heated to 60 °C, followed by dropwise addition of hydrazine hydrate (85%, 2.5 mL). Upon completion of addition, the reaction was maintained at 60 °C for 1 h until the solution turned orange-red. After cooling to room temperature, the pH was adjusted to 7 using 6 mol·L^−1^ HCl. The mixture was refrigerated (0–4 °C) overnight, then filtered and washed with water. The crude product was recrystallized from water (100 °C) to afford 1-amino-2-nitroguanidine as a white solid (2.38 g).

#### 3.3.3. 1-Amino-2-nitroguanidinium Nitrate (ANGN)

To a vigorously stirred suspension of derivative 3 (1.4 g, 0.01 mol) in water (25 mL) was added 40% nitric acid (10 mL, 0.02 mol). The solution was stirred at 60 °C for 10 min and then cooled to room temperature. IR ν/cm^−1^: 3391, 1644, 1574, 1463, 1337, 1197, 911, 822. δ_H_ (DMSO-*d*_6_, 500 MHz): 9.78 (s, 1H), 8.67 (s, 1H); δ_C_ (DMSO-*d*_6_, 126 MHz): 159.3; δ_N_ (DMSO-*d*_6_, 51 MHz): −6.6, −15.0. *m*/*z* (ESI): 120.08 [M + H]^+^, 62.08 [M-H]^−^.

### 3.4. Calculation Details

In this work, the crystal structures of NQ, ANQ, and ANGN were obtained from the CCDC database. All calculations were carried out using Gaussian 16 (A.03) software [27], and all analyses, including electrostatic potential (ESP), Hirshfeld surface (HS), and the independent gradient model based on Hirshfeld partition (IGMH) analyses, were performed by the wavefunction analysis Multiwfn 3.8 tools [28,29,30,31,32] and visualized with Visual Molecular Dynamics (VMD) [33]. To represent the molecular states in the crystal environment as well as possible, the heavy atoms C, N, and O were kept fixed, and only H atoms were optimized under constraints at the B3LYP-D3/6-311+G(d,p) level [34,35,36,37,38,39,40]. These wavefunctions of supramolecular architectures of these compounds were produced at the B3LYP-D3/6-311+G(d,p) level, and the wavefunctions of other analyses were produced using M062X-D3 in conjunction with the def2-QZVPP basis set [41,42]. The energy decomposition analyses (EDA) were performed on the dimer complexes of NQ, ANQ, and ANGN [25].

## 4. Conclusions

In summary, this work systematically discusses the structural features, thermal decomposition, and sensitivity of NQ, ANQ, and ANGN and elucidates the underlying reasons for the variations in their density, thermal decomposition, and sensitivity. The study provides unique insights into the intrinsic mechanisms controlling the variations in density, sensitivity, and thermal decomposition of nitroguanidine derivatives.

(1) The charged nature of ANGN enhances the hydrogen bonding network via stronger electrostatic and inductive interactions, leading to its higher density. In contrast, the lower maximum value of the positive electrostatic potential in ANQ weakens the hydrogen bonding network due to diminished electrostatic interactions, resulting in a lower density. These findings are consistent with the observed trend in crystal density: ANGN > NQ > ANQ.
(2) The distribution of electrostatic potential of ANQ and ANGN becomes more inhomogeneous due to the introduction of -NH_2_ groups and NO_3_^−^ anions, resulting in an increase in sensitivity. Furthermore, the frequency of O···O contacts and the strength of the hydrogen bonding network are also closely consistent with the experimental sensitivity order: ANGN > ANQ > NQ.
(3) LOL-π analysis reveals that the order of molecular conjugation strength is ANQ > NQ > ANGN due to the differences in the electronic effects of introduced groups, which aligns with the trend in N–NO_2_ bond dissociation energy and the corresponding yield of NO_2_ during decomposition. Furthermore, the trend in HOMO-LUMO gaps (NQ > ANQ > ANGN) successfully explains the thermal behavior: a larger gap can lead to a higher decomposition temperature.

## Figures and Tables

**Table 1:** Physical and energetic properties of NQ, ANQ, and ANGN compared with RDX and HMX.

	NQ	ANQ	ANGN	RDX	HMX	CL-20
OB ^a^/[%]	−30.8	−33.6	0.0	−21.6	−21.6	11.0
ρ ^b^/[g·cm^−3^]	1.76	1.72	1.85	1.80	1.91	2.04
∆_f_H ^c^/[kJ·mol^−1^]	−86.0	76.9	15.5	71.1	74.0	377
V_D_ ^d^/[m·s^−1^]	8629	9096	9438	8794	9198	9754
P ^e^/[GPa]	27.7	31.1	40.2	33.7	38.2	44.9
Q ^f^/[kJ·kg^−1^]	3563	4631	6230	5715	5671	6172
I_sp_ ^g^/[s^−1^]	209.2	238.2	274.4	266.5	265.1	270.8
f ^h^/[kJ·kg^−1^]	980.7	1243	1361	1385	1365	1313
T_d_ ^i^/[°C]	254	184	148	204	275	215
IS ^j^/[J]	>50	20	10	7.4	7.4	4
FS ^k^/[N]	>355	144	120	120	120	48
Ref ^l^	[19]	[18]	[14]	[20]	[20]	[21]

^a^ Oxygen balance. ^b^ Crystal density. ^c^ Heat of formation. ^d^ Detonation velocity. ^e^ Detonation pressure. ^f^ Heat of detonation. ^g^ Specific impulse. ^h^ Gunpowder impetus. ^i^ Decomposition temperature. ^j^ Impact sensitivity. ^k^ Friction sensitivity. ^l^ Reference.

## Data Availability

All additional data are available in the Supplementary Materials file.

## Supplementary Materials

The following supporting information can be downloaded at https://www.mdpi.com/article/10.3390/molecules30214204/s1: Figure S1: IR spectra of NQ, ANQ, and ANGN; Figure S2: ^1^H NMR spectrum of NQ in DMSO-*d*_6_; Figure S3: ^13^C NMR spectrum of NQ in DMSO-*d*_6_; Figure S4: ^1^H NMR spectrum of ANQ in DMSO-*d*_6_; Figure S5: ^13^C NMR spectrum of ANQ in DMSO-*d*_6_; Figure S6: ^1^H NMR spectrum of ANGN in DMSO-*d*_6_; Figure S7: ^13^C NMR spectrum of ANGN in DMSO-*d*_6_; Figure S8: TG-DSC curves of NQ (**a**), ANQ (**b**), and ANGN (**c**) at a heating rate of 10 °C min^−1^; Figure S9: NPA charges of NQ, ANQ, and ANGN; Figure S10: HOMO-LUMO gaps of NQ, ANQ, and ANGN; Figure S11: Hirshfeld finger-print maps of NQ, ANQ, and ANGN; Figure S12: Gradient isosurfaces of 1 (**a**), 2 (**b**), and 3 (**c**). Scatter graph between δ_g_^inter/intra^ and sign(λ_2_)*ρ* of 1 (**d**), 2 (**e**), and 3 (**f**). Red: δ_g_^inter^; dark gray: δ_g_^intra^. Table S1: Hydrogen bonds formed by a molecule in the crystal structure of NQ, ANQ, and ANGN.
