# Supplementary material for: Unraveling the Intrinsic Mechanisms Controlling the Variations in Density, Sensitivity, and Thermal Decomposition of Typical Nitroguanidine Derivatives
Source: Molecules. 2025 Oct 28;30(21):4204. doi: 10.3390/molecules30214204 (PMC12608654; doi:10.3390/molecules30214204)
Supplement: Supplementary file 1 [file molecules-30-04204-s001.zip › molecules-3934007-supplementary.pdf]

# Supplemental Information

## Unraveling the Intrinsic Mechanisms Controlling the Varia-tions in Density, Sensitivity, and Thermal Decomposition of Typical Nitroguanidine Derivatives

**Pengshan Geng [1], Songsong Guo [1], Xiaohong Wang [1], Chao Xing [1], Chenxi Qu [1], Jieyu Luan [1] and Kewei Ding [1,2*]**

[1] Xi'an Modern Chemistry Research Institute, Xi'an 710065, China

[2] State Key Laboratory of Fluorine & Nitrogen Chemical, Xi'an 710065, China

[*] Correspondence: dkw204@163.com

## Table of Contents

1. IR spectra ········ S3
2. $^{1}$H and $^{13}$C NMR spectra ········ S3
3. TG-DSC ········ S6
4. NPA charge ········ S7
5. HOMO-LUMO gap ········ S7
6. Hirshfeld finger-print maps ........ S7
7. IGMH analysis ........ S8
8. The details of hydrogen bonds ........ S8

1. IR spectra

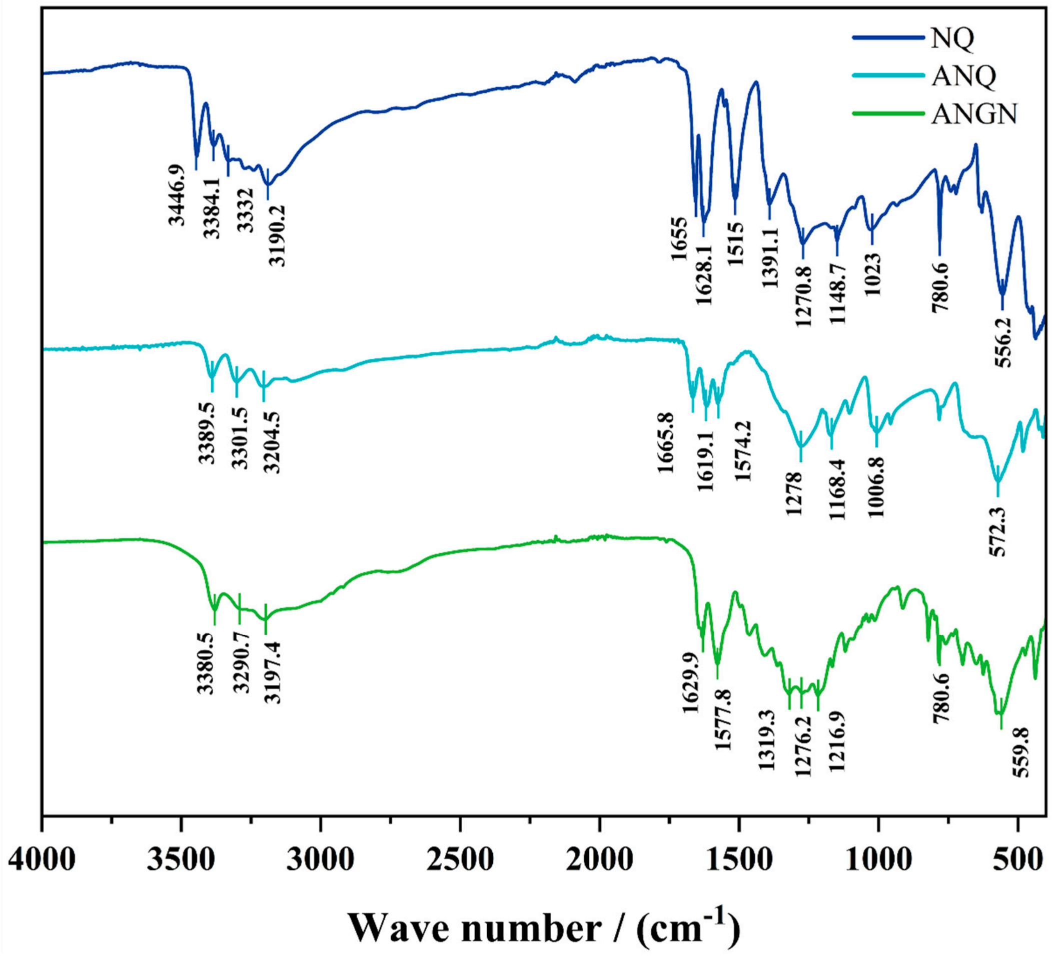


**Figure S1**. IR Spectrum of NQ, ANQ and ANGN.

2. $^{1}H$ and $^{13}C$ NMR spectra

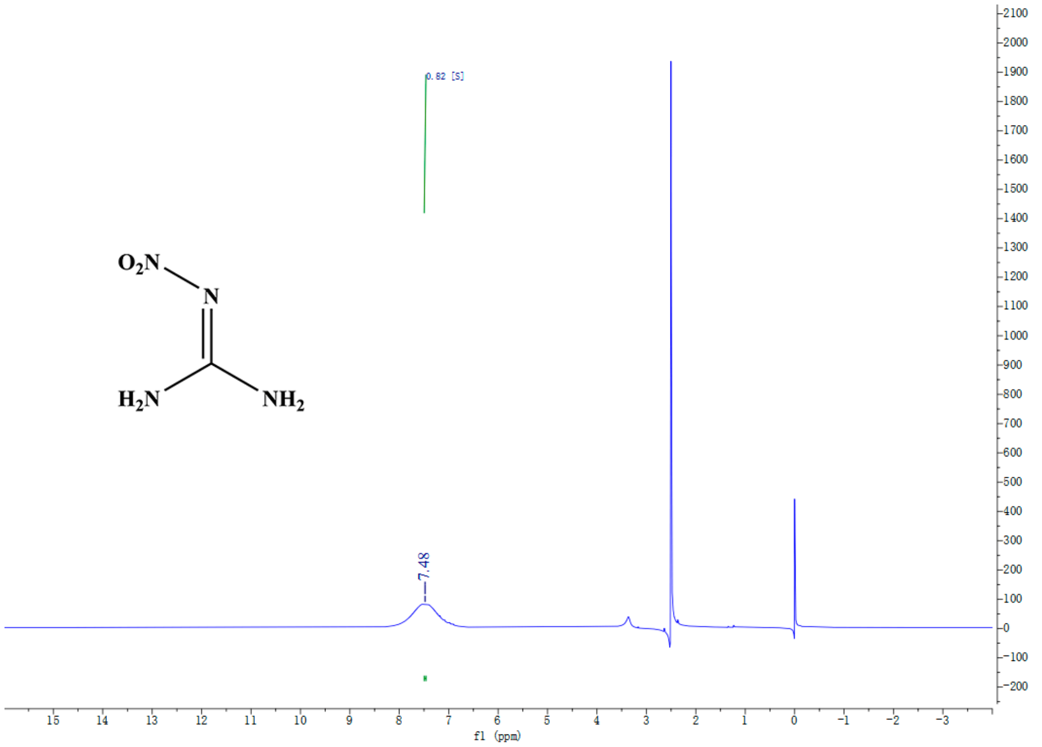


**Figure S2.** $^{1}$H NMR Spectrum of NQ in DMSO-*d*6.

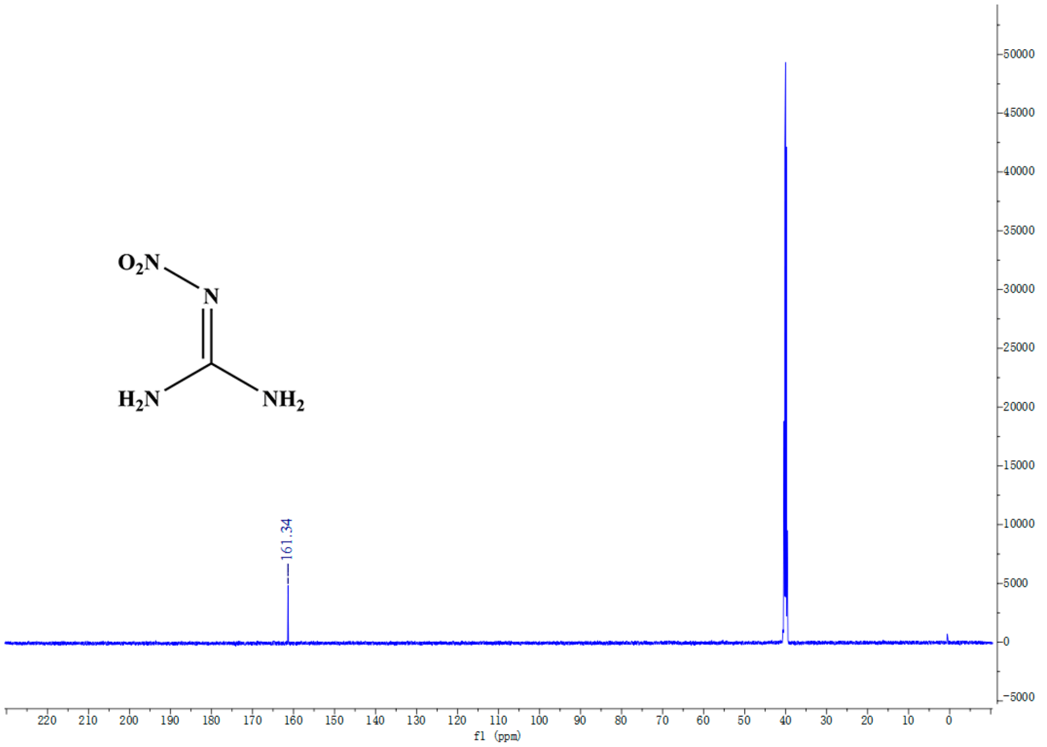


**Figure S3**. $^{13}$C NMR Spectrum of NQ in DMSO-*d*6.

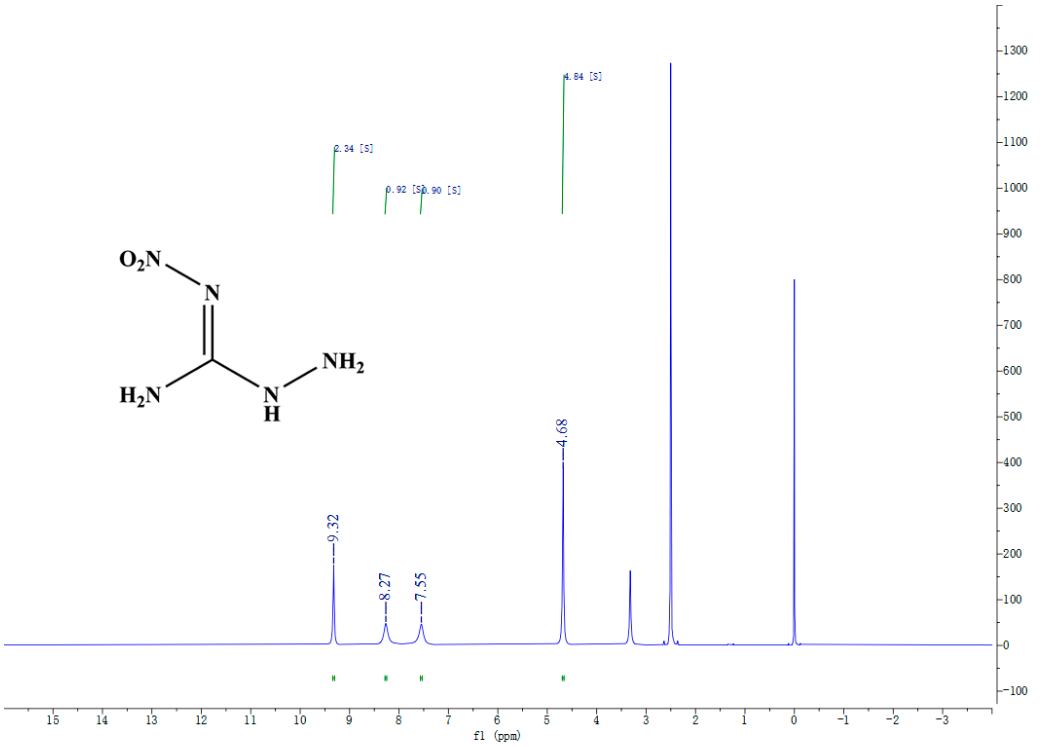


**Figure S4.** $^{1}$H NMR Spectrum of ANQ in DMSO-$d_6$.

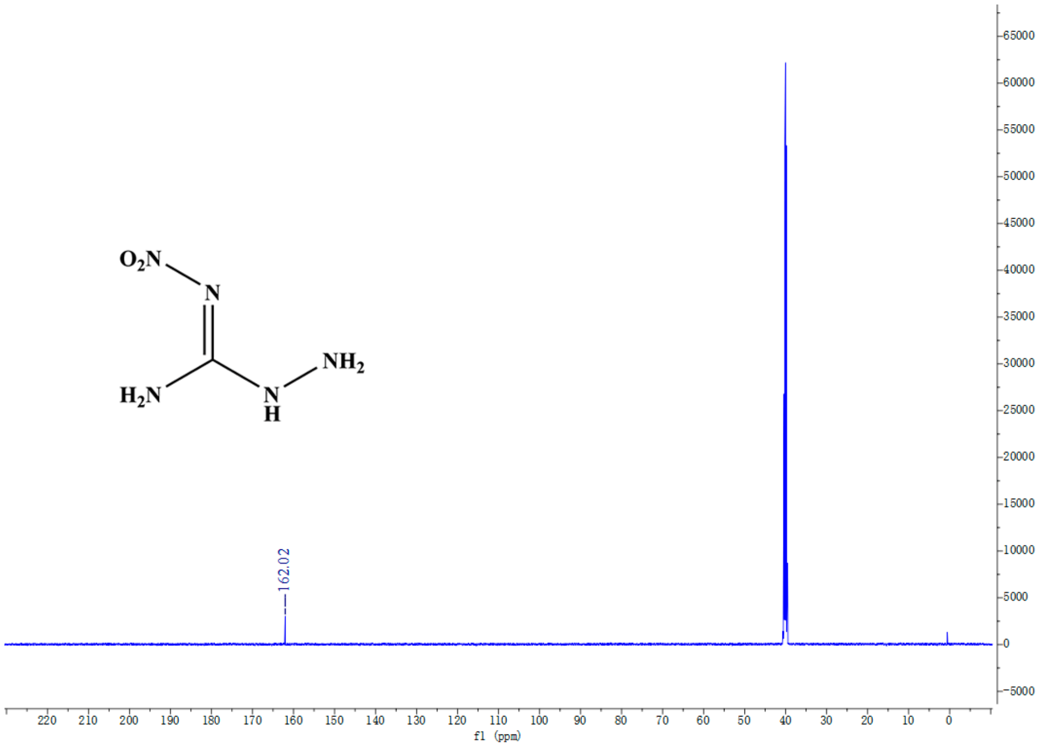


**Figure S5**. $^{13}$C NMR Spectrum of ANQ in DMSO-$d_6$.

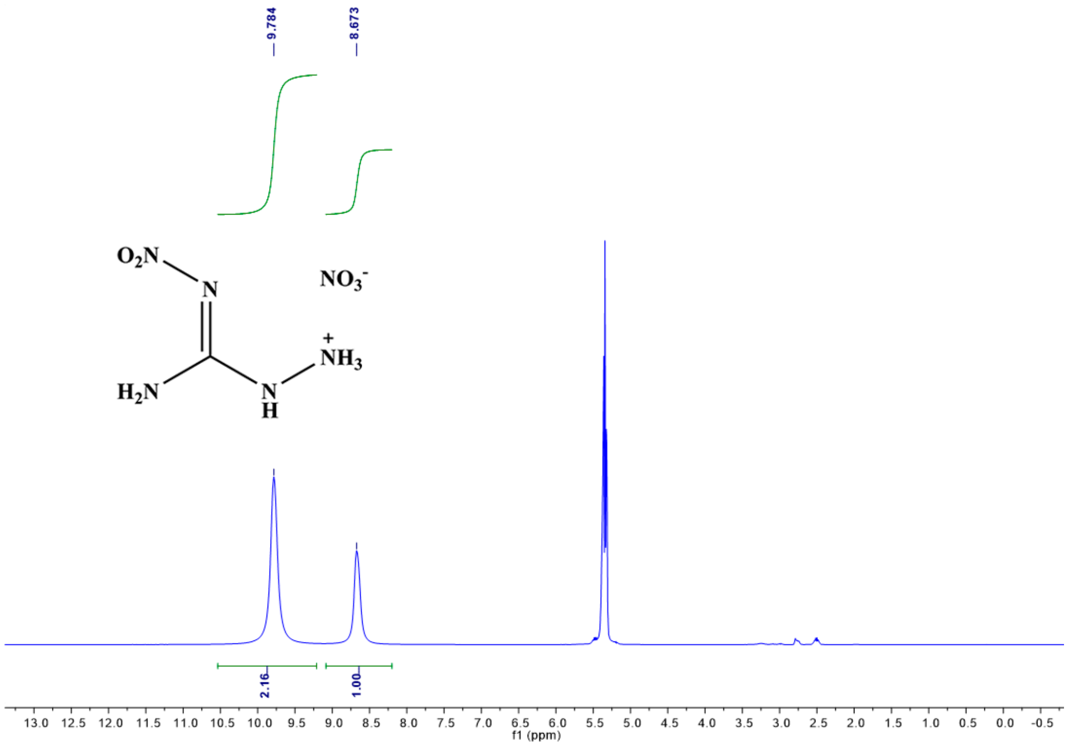


**Figure S6.** $^1H$ NMR Spectrum of ANGN in DMSO-$d_6$.

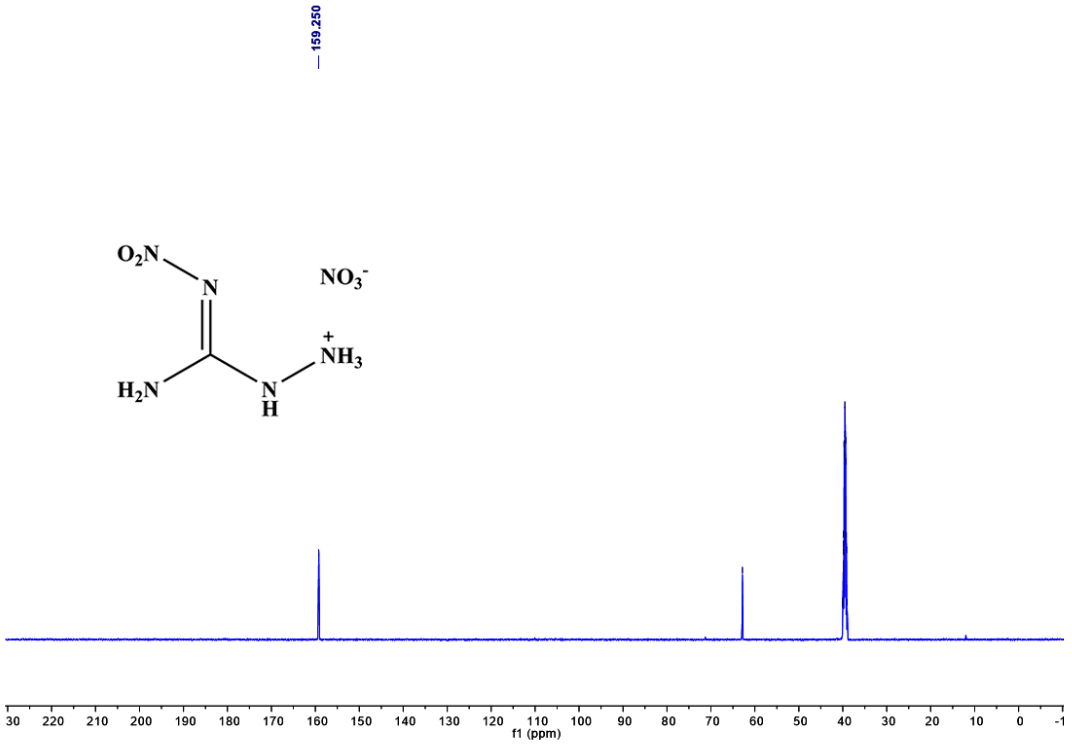


**Figure S7**. $^{13}C$ NMR Spectrum of ANGN in DMSO-$d_6$.

## 3. TG-DSC

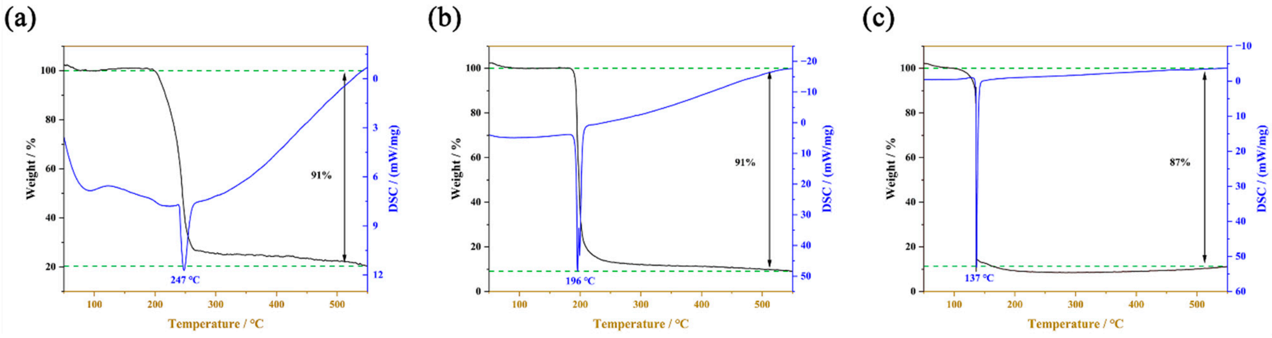


**Figure S8**. TG-DSC curves of NQ (a), ANQ(b) and ANGN(c) at the heating rate of 10 °C min⁻¹.

## 4. NPA charge

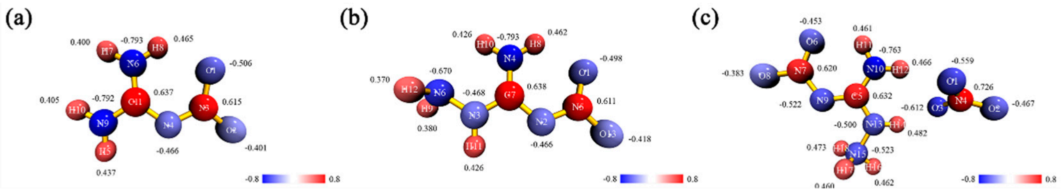


**Figure S9**. NPA charges of NQ, ANQ and ANGN.

## 5. HOMO-LUMO gap

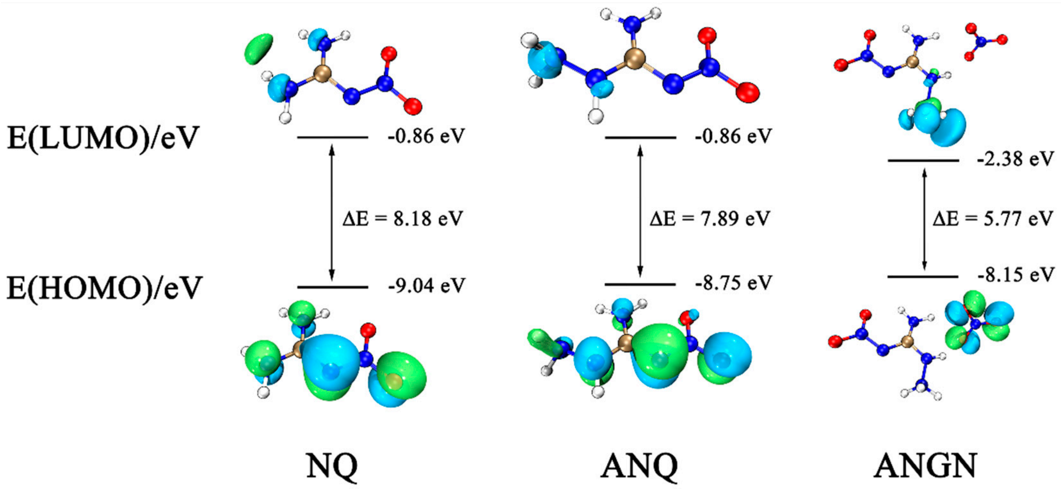


**Figure S10.** HOMO-LUMO gaps of NQ, ANQ and ANGN.

## 6. Hirshfeld finger-print maps

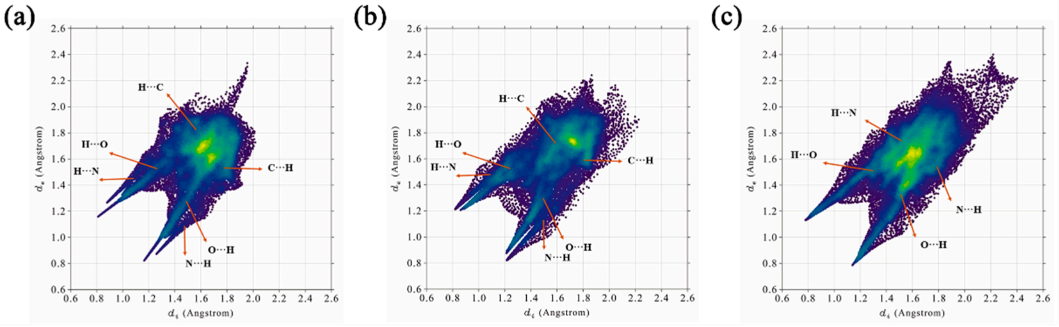


**Figure S11.** Hirshfeld finger-print maps of NQ, ANQ and ANGN.

## 7. Hirshfeld finger-print maps

In the IGMH isosurfaces (Figure S12a-c), the sites and strengths of hydrogen bonds can be clearly observed. For ANQ, the introduced $NH_2$ group provides additional hydrogen bond donors to form more hydrogen bonds. Different from ANQ, ANGN forms stronger hydrogen bonds though hydrogen bond acceptors in the nitrate anion. Furthermore, the scatter graphs (Figure 12d-f) reveal that the intensity of their hydrogen bonding network.

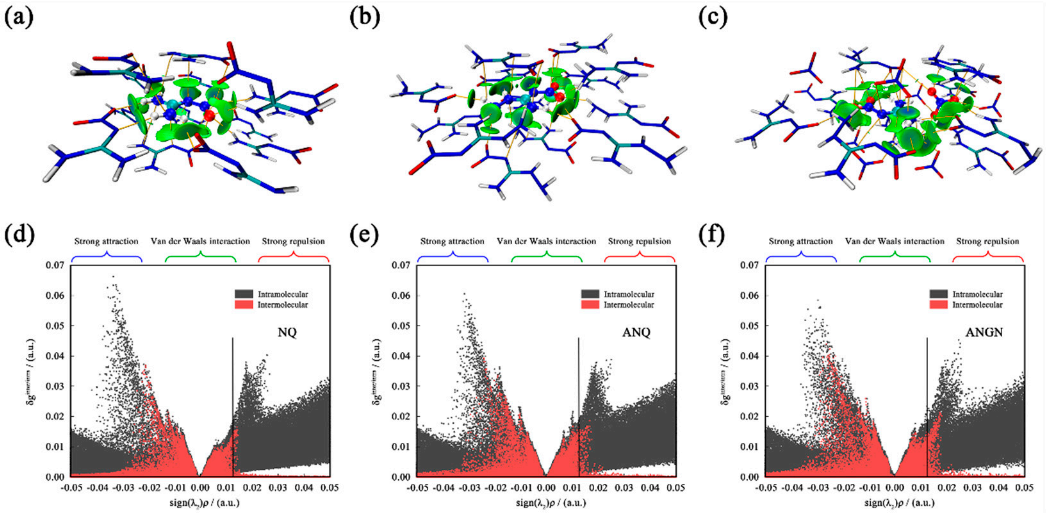


**Figure S12.** Gradient isosurfaces of 1 (a), 2 (b), and 3 (c). Scatter graph between $\delta_g^{inter/intra}$ and sign($\lambda_2$)ρ of 1 (d), 2 (e), and 3 (f). red: $\delta_g^{inter}$; darkgray: $\delta_g^{intra}$.

## 8. The details of hydrogen bonds

The atomic number is consistent with that shown in Figure S9.

Table S1. Hydrogen bonds formed by a molecule in the crystal structure of NQ, ANQ and ANGN.

| NQ | $r^a$/Å | $BE^c$/kcal·mol$^{-1}$ | ANQ | $r^a$/Å | $BE^c$/kcal·mol$^{-1}$ | ANGN | $r^a$/Å | $BE^c$/kcal·mol$^{-1}$ |
|---|---|---|---|---|---|---|---|---|
| O2···H | 1.986 | -11.72 | O13···H-N | 2.103 | -8.99 | O3···H | 1.967 | -33.91 |
| H10···O | 1.969 | -11.68 | N2···H | 2.034 | -8.92 | H16···O | 1.973 | -33.81 |
| H7···N | 2.143 | -10.69 | O117···H | 2.087 | -8.91 | O3···H | 2.031 | -26.08 |

| | | | | | | | | |
|---|---|---|---|---|---|---|---|---|
| H8···O | 2.243 | -8.73 | H12···O | 2.068 | -7.72 | O1···H | 2.025 | -23.42 |
| O1···H | 1.931 | -8.64 | O1···H | 2.172 | -7.56 | H17···O | 1.909 | -22.37 |
| N4···H | 2.131 | -8.59 | H11···N | 2.031 | -7.20 | H18···O | 1.950 | -22.23 |
| H5···O | 2.319 | -6.66 | O117···H | 2.103 | -3.27 | O1···H | 2.049 | -20.83 |
| O1···H | 2.317 | -6.46 | H8···O | 2.107 | -3.25 | O2···H | 1.971 | -19.75 |
| O2···H | 2.497 | -6.28 | N6···H | 2.242 | -1.56 | O8···H | 1.925 | -19.67 |
| | | | H10···N | 2.243 | -1.56 | | | |

| Type of energy | kcal·mol$^{-1}$ | Type of energy | kcal·mol$^{-1}$ | Type of energy | kcal·mol$^{-1}$ |
|---|---|---|---|---|---|
| **Total bond energy** | **-79.45** | **Total bond energy/kcal·mol$^{-1}$** | **-58.93** | **Total bond energy/kcal·mol$^{-1}$** | **-227.96** |
| **Average bond energy** | **-8.83** | **Average bond energy/kcal·mol$^{-1}$** | **-5.89** | **Average bond energy/kcal·mol$^{-1}$** | **-22.80** |
| **Average electrostatic energy** | **-8.70** | **Average electrostatic energy** | **-6.88** | **Average electrostatic energy** | **-19.53** |
| **Average dispersion energy** | **-3.59** | **Average dispersion energy** | **-3.95** | **Average dispersion energy** | **-5.66** |
| **Average induction energy** | **-2.90** | **Average induction energy** | **-3.25** | **Average induction energy** | **-8.84** |
| **Average exchange-repulsion energy** | **6.36** | **Average exchange-repulsion energy** | **8.19** | **Average exchange-repulsion energy** | **11.22** |
